# Gleanings from Our Exchanges

**Published:** 1874-05-15

**Authors:** 


ARTIFICIAL REST IN PLEURISY.—Dr. Roberts says, in the *Practitioner:* In the *early* stage of the disease I would strongly recommend that a trial should be given to the plan of *mechanically fixing the entire side* by one of the methods to be now described. In order to be of any use it should be done effectually, so as to restrain the movements as much as possible, and the sooner the application is made, the more likely is it to be of service. The plan I originally adopted was the following: Strips of adhesive plaster, from four to five inches wide, were fixed at one end, close to the spine, and then drawn tightly round the side, as far as the middle line in front, the patient being directed to expire deeply. In this manner the whole side was included, commencing from below and proceeding upward, each succeeding strip partially overlapping the one below. One was also fixed over the shoulder. Over this layer of plaster strips of bandage of the same width were fixed in like manner, having been previously dipped in a mixture of mucilage and chalk, such as is used in the treatment of fractures. Two or three layers of these were laid on, and then heated sand-bags applied, in order to dry the application as soon as possible. This is a most effectual mode of fixing one side of the chest, while it leaves the other quite free to act; and I would, by the way, commend it to those who are called upon to treat fractured ribs. The plaster adheres firmly to the skin, and the bandages adhere to the plaster, a firm casing being formed which will remain on any length of time. With regard to pleurisy, however, I have since then adopted another plan, which, so far as the disease is concerned, seems sufficiently efficacious. It is merely to use strips of plaster, putting on two or three layers in the following manner: The first strip is laid on obliquely *in the direction of the ribs*, the second *across the course of the ribs*, the third in the direction of the first, about half overlapping it, the fourth the same as the second, and so on until the entire side is covered. A strip is also passed over the shoulder, which is kept down by another fixed round the side across its ends. Now it is difficult positively to prove that this treatment actually checks the course of pleurisy; but, taking a common sense view of the matter, it is not improbable that such a result is anticipated; and, from my own experience, I have not the slightest doubt but that it is brought about. I have carried it out now in a good number of cases, and in all the course and termination have been most satisfactory, while relief to the pain and other distressing symptoms has been generally immediate. I feel convinced, also, that in many of those cases of extensive pleuritic effusion which come under observation, the accumulation might have been prevented or moderated had this plan of treatment been adopted at an early period.—*Philadelphia Med. and Surg. Reporter.*

---

TOLERANCE OF THE HEART TO TRAUMA.—The following case, translated from the *Gazette des Hopitaux*, we clip from *The Clinic* of May 2d:

An old soldier was received during the early part of March, in the Hotel-Dieu, service of Richet, a few moments after an attempt at suicide. He had discharged a revolver upon the region of the heart. The ball entered below the left nipple, and did not escape, but made a track behind, by the side of the vertebral column, where it seemed to be lodged. The wound occasioned very little inconvenience and almost no dyspnœa. So little was his distress that the internes believed that the ball had not penetrated, but had simply traversed the circumference of the thoracic cavity. A careful examination, with the *esprit* of M. Richet, led to a contrary conviction. He recognized, in fine, by percussion, practiced with great caution, dullness at the presumed level of the projectile. The summit of the left lung yielded a tympanitic resonance, and the ear applied to the chest perceived coarse mucous rales with the metallic bruit. Finally, there supervened some expectoration of pure blood, which could leave no doubt of the affection of the lung. The lung had been thus traversed, and there was left, most probably, hæmato-pneumo-thorax.

As to the heart, it beat with its ordinary regularity. Nevertheless, M. Richet believed that he must maintain reserve as to a possible lesion of this organ, the orifice of the entry of the ball being at the level of the apex.

M. Richet prescribed blood-letting copiously, *coup sur coup*, iced drinks, and internal hæmostatics.

On the next morning the patient was seized with an attack of cough, which was followed by a sharp hæmorrhage from the wound. With every movement of inspiration and expiration, there escaped a considerable quantity of frothy blood, with the bubbling discharge of air. At the same time there developed emphysema of all the upper parts of the body. The heart remained always regular, as if impassive, as also the pulse. Death followed in the afternoon from the continued hæmorrhage which nothing could arrest.

At the autopsy was recognized a fracture of the rib at the level of the entrance of the ball. The path of the ball traversed the pleura and the pericardium successively at the level of the apex of the heart, which was the seat of a small contused wound. About a table-spoonfull of clotted blood laid in the pericardium. About the wound could be seen the traces of an extensive contusion of the surface of the heart, produced, without doubt, by friction against the fragments of the rib. The left inferior lobe of the lung was traversed in its whole extent.

---

OBITUARY.—In Wiesbaden, Feb. 19, 1874, of apoplexy, in his 65th year, Dr. Karl Ernst Bock, Professor of Pathological Anatomy at Leipsic, for years associate editor of *Schmidt's Jahrbucher*, and during his whole life a most zealous worker in his department of medical science.

---

SPECIAL RULES for the management of infants during the hot season, recommended by the Obstetrical Society of Philadelphia, 1874,

As worthy of special notice, we extract the last rule:

RULE 11.—Do not wean the child just before or during the hot weather; nor, as a rule, until after its second summer. If suckling disagrees with the mother, she must not wean the child, but feed it in part, out of a nursing-bottle, on such food as has been directed. However small the supply of breast-milk, provided that it agrees with the child, the mother should carefully keep it up against sickness; it alone will often save the life of a child when everything else fails. When the child is over six months old, the mother may save her strength by giving it one or two meals a day of stale-bread and milk, which should be pressed through a sieve and put into a nursing-bottle. When from eight months to a year old, it may have also one meal a day of the yolk of a fresh and rare-boiled egg, or one of beef or mutton-broth, into which stale-bread has been crumbed. When older than this, it can have a little meat finely minced; but even then milk should be its principal food, and not such food as grown people eat.—*The Clinic.*

---

DESTRUCTION OF BRAIN SUBSTANCE WITHOUT FUNCTIONAL LESION.—Prof. Porta, of Pavia, gives an account (*Archivio Italiano*, November, 1873; abstr. in *Psychiatr. Centralblatt*) of the case of a man who had received an injury of the skull, causing, as nearly as could be estimated, the complete disorganization of the upper right hemisphere. In spite of this extensive lesion, no measurable psychic or sensorial disturbance was observed; and at the end of eighteen months a partial hemiplegia of the left side only, remained. This was apparently somewhat improved by electrical treatment.

The same author reports another case of the *post-mortem* of a woman who had died of fever, without stupor, somnolence, or delirium, in whom the whole right side of the brain was found disorganized by suppuration, the only parts remaining intact being the cerebellum, the pons, the crus cerebelli, and the intraventricular portion.

From these facts Prof. Porta holds that the brain is a double organ, consisting of two similar halves, one of which can do the duty of both; that is, that it is, physiologically, as well as anatomically, double.—*Chicago Jour. of Nervous and Mental Disease.*

